# A Review on Pathological and Diagnostic Aspects of Emerging Viruses—*Senecavirus A*, *Torque teno sus virus* and Linda Virus—In Swine

**DOI:** 10.3390/vetsci9090495

**Published:** 2022-09-10

**Authors:** Salwa Hawko, Giovanni P. Burrai, Marta Polinas, Pier Paolo Angioi, Silvia Dei Giudici, Annalisa Oggiano, Alberto Alberti, Chadi Hosri, Elisabetta Antuofermo

**Affiliations:** 1 Department of Veterinary Medicine, University of Sassari, 07100 Sassari, Italy; 2 Department of Animal Health, Istituto Zooprofilattico Sperimentale della Sardegna, 07100 Sassari, Italy; 3 Department of Veterinary Medicine, Faculty of Agronomy and Veterinary Sciences, Lebanese University, Beirut 14/6573, Lebanon

**Keywords:** emerging virus, pigs, gross lesions, histopathology, diagnoses

## Abstract

**Simple Summary:**

Worldwide demand for food is expected to increase due to population growth and swine accounts for more than one-third of meat produced worldwide. Several factors affect the success of livestock production systems, including animal disease control. Despite the importance of infectious diseases to animal health and the productivity of the global swine industry, pathogens of swine, in particular emerging viruses, such as *Senecavirus A*, *Torque teno sus virus*, and Linda virus, have gained limited interest. We performed a systematic analysis of the literature, with a focus on the main macroscopical and histological findings related to those viruses to fill the gap and highpoint these potentially hazardous pathogens.

**Abstract:**

Swine production represents a significant component in agricultural economies as it occupies over 30% of global meat demand. Infectious diseases could constrain the swine health and productivity of the global swine industry. In particular, emerging swine viral diseases are omnipresent in swine populations, but the limited knowledge of the pathogenesis and the scarce information related to associated lesions restrict the development of data-based control strategies aimed to reduce the potentially great impact on the swine industry. In this paper, we reviewed and summarized the main pathological findings related to emerging viruses, such as *Senecavirus A*, *Torque teno sus virus*, and Linda virus, suggesting a call for further multidisciplinary studies aimed to fill this lack of knowledge and better clarify the potential role of those viral diseases in swine pathology.

## 1. Introduction

Swine livestock represents a significant source of meat for human consumption since it occupies over 30% of global meat demand [1]. Pork production reached a level where it accounts for more than one-fourth of total protein consumed worldwide [1], as over 90 million metric tons of pork are produced globally on an annual basis [2]. In Europe, the pork industry accounts for about 150 million reared pigs, representing nearly half of the total EU production of meat [3]. This production places the EU as the world’s second-biggest pork producer after China and the first exporter of pork products, especially after the fall in the production of pork in Asia, caused by African Swine Fever [4].

In Italy, pig farms are mainly localized in Northern Italian regions (Lombardia, Emilia-Romagna, Piemonte, Veneto), with livestock of more than 9 million pigs, representing 6% of the agri-food industry [3,5]. From 1960, swine farms shifted from small farms to intensive rearing systems [6]. Although, according to Espinosa and colleagues, the spread of pathogens within intensive farms is less frequent, the risk of contamination during transportation, the susceptibility to immunosuppression, and the rapid spread of diseases could be increased [7]. One of the main threats to production in intensive pig farms is infectious agents, such as bacteria and viruses. Historically, viral diseases that had a great negative impact on the swine industry causing great economic losses are Porcine Reproductive and Respiratory Syndrome (PRRS), porcine circovirus-associated diseases (PCVAD), African Swine Fever (ASF), and the Foot and Mouth Disease (FMD) [4,8,9,10,11,12].

PRRS was first described in Canada in the late 1980s and later spread in the US, Asia, and EU countries [9], causing mild to severe respiratory disease in newborn infected piglets and reproductive failure in pregnant sows [9]. Porcine Circovirus 2 (PCV2), discovered in 1998, is also among the most important viruses affecting pig production, leading to lymphoid depletion, histiocytic infiltration, giant cells with botryoid inclusions, and immunosuppression in pigs. It causes several associated diseases, known as porcine circovirus-associated diseases [10,11]. ASFV was first identified in Kenya, in 1921, has remained endemic in Africa and spread in many countries, causing symptoms ranging from chronic, subclinical to hemorrhagic fever, and peracute death [12]. FMD is a highly contagious viral disease of livestock that has a significant economic impact, causing lameness, anorexia, and vesicles on the feet and mouth [13].

Recently, novel emerging viruses in pigs, such as porcine circoviruses 3 and 4 (PCV3, PCV4), *Senecavirus A* (SVA), parvoviruses (PPV) 2, 3, 4, 5, 6, 7, *Torque Teno Sus virus* (TTSuV), atypical porcine pestivirus (APPV), lateral-shaking inducing neuro-degenerative agent (Linda) virus, porcine deltacoronavirus (PDCoV), and swine enteric alphacoronavirus (SEACoV), appeared and significantly impacted on this sector worldwide, since they can have a wide diffusion [14,15]. Adding to this, as underlined by Meng in 2012 and, more recently, by Perfumo and colleagues in 2020, emerging viruses have no country boundaries, could quickly spread, and pose a new challenge for researchers and veterinarians [14,15].

Emerging viral diseases are believed to be associated with a change in the cycle of agent, host, and environment and these changes are linked to several factors, such as mutations or recombination of the agent’s RNA or ssDNA, the presence of a pathogen into a new host, the capacity of a pathogenic agent to multiply and spread in a healthy herd, and unprofessional veterinary activities [15]. Nevertheless, the effect of coexisting infections due to multiple pathogens and the efficacy of modified live attenuated vaccines needs further investigation and studies [16].

Despite the majority of these viruses being omnipresent in swine populations worldwide, they are extremely understudied with a lack of knowledge on pathological lesions in natural and experimental infection as well as diagnostic methods used to detect emerging viruses [14,15]. Nevertheless, the low level of awareness regarding the pathogenesis and the related lesions as well as the available diagnostic methods pitfall the risk management assessments and limit the development of control strategies aimed to reduce the potentially great impact on the swine industry.

Among emerging viruses, *Senecavirus A* poses a major concern because of the similarity with other swine diseases, particularly with FMD, a World-Organisation-for-Animal-Health-listed diseases. On the other hand, *Torque teno sus viruses* could trigger the development of disease by important porcine viral pathogens, such as PCV2, PPV, and PRRSV. Linda virus, a newly proposed Pestivirus that is claimed to cause congenital tremors and preweaning mortality, has a loco-regional distribution in Styria, Austria, and could have implications for classical swine fever virus surveillance and porcine health management.

This review focuses on pathogenic potential, gross lesions, histologic features, and the main diagnostic methods of the selected emerging swine viruses (*Senecavirus A*, *Torque teno sus virus*, and Linda virus) that have uncertain clinical pathological significance on pig health, suggesting a call for further multidisciplinary studies aimed to fill this lack of knowledge and better clarify the potential role of those viral diseases in swine pathology.

## 2. *Senecavirus A*

*Senecavirus A* (SVA), also known as Seneca Valley virus (SVV), is a non-segmented, non-enveloped, icosahedral, linear positive-sense, single-stranded RNA, belonging to the family *Picornaviridae*, and is the only virus in the genus *Senecavirus* [17].

SVA genome has a length of approximately 7.2 kb and its capsid is about 30 nm in diameter [17,18]. This genus presents the standard layout of *Picornavirus* composed of a leader (L) and several polypeptides divided into P1 (four polypeptides), P2 (three polypeptides), and P3 (four polypeptides) [17,18]. The capsid contains 60 protomers with four structural proteins VP1, VP2, VP3, and VP4 for each protomer with a length of 263, 284, 239, and 72 residues [19]. The RNA genome of SVA contains about 7300 nucleotides and one single large open reading frame (ORF), encoding for a polyprotein of 2181 amino acids; it is flanked by 5′ untranslated region (UTR) and 3′ UTR. At the 5′ UTR, a type IV internal ribosome entry site (IRES) is located, which promotes translation of the viral RNA by inhibiting the translation of cellular RNA and is closely similar to the one in Hepatitis C and Classical swine fever [17,18]. The 5′UTR is 666 bases long, whereas the 3′UTR is shorter and it is 70 bases long and contains a polyadenylated tail. After the translation of this RNA genome into one polyprotein, it is cleaved by viral proteases into four mature proteins (VP1, VP2, VP3 located in the outer capsid and VP4 located in the internal) and eight non-structural proteins (2A, 2B, 2C, 3A, 3B, 3C, 3D, L) [17,18,19,20].

The *Seneca Valley virus* (SVV-001) was detected as a contaminant in a human retinal cell culture PER.C6 (transformed cell retinoblast) in 2002 at Genetic Therapy Inc. in Gaithersburg, MD, USA, close to the Seneca Valley geographic region [17]. Furthermore, the sequencing of picorna-like viruses isolated from affected pigs revealed the presence of SVA in the USA since the late 1980s [21].

Despite that, *Senecavirus A* was detected by PCR in 2007 from vesicular lesions on the snout of a pig that arrived at a harvest facility in Minnesota, with a trailer load of 187 Canadian market hogs [22]. However, the detailed description of gross and histological lesions, as well as the tentative that led to SVA being considered as an etiological agent of a vesicular disease, was reported by Singh and coauthors in 2012 in a 6-month-old intact male Chester White boar with a history of anorexia, lethargy, and lameness. Intact and ruptured vesicles and erosions were observed in the oral cavity, around the nares and the coronary bands, and ulcers were detected on the forelimbs and hind limbs [23].

Of interest, in 2014, various acute swine disease outbreaks of SVA were discovered by next-generation sequencing and RT-PCR in Brazil and were characterized by: (i) the presence of vesicles and coalescing erosions on the snouts and coronary bands of sows, (ii) acute loss of neonatal piglets (30–70%) in the first four days of age, and (iii) self-limiting outbreaks lasting approximately 1–2 weeks [24]. Some herds suffered an increase in neonatal losses (30–70%) named epidemic transient neonatal losses (ETNL). The detected strains shared a 94.2–96.5% nucleotide identity with the known SVV-001 genome [24]. Interestingly, no circulating antibodies were detected before 2014 in Brazil’s major swine-producing states [25].

In 2015, several cases of vesicular disease characterized by acute lameness and coronary band vesicles without mortality were observed in exhibition swine and breeding herds in Iowa [26,27]. Additional cases have since been recognized in Iowa and Ohio [28,29].

The first reports of SVA in China were described in two farms where animals showed vesicular lesions and lameness in 2015. The detected strain of the SVA was similar to the United States and Brazilian isolates [30,31].

In the following years, SVA progressively disseminated into Chinese provinces, with more than half of the provinces, autonomous regions, and municipalities affected by SVA infection. As a result of phylogenetic analysis, China isolates were grouped into five genetic branches [32], with evidence of genetic recombination among strains [33,34].

In February 2016, SVA-associated vesicular lesions were observed in Colombia and the detected strains shared a 98.5–98.9% nucleotide homology with the previously documented strains in the US (KX857728) [35].

In October 2016, vesicular lesions were described in swine in Thailand, with 98.2% sequence homology to the Canadian strain 11-55910-3 [36]. SVA-associated vesicular lesions appeared in Vietnam in 2018 and isolated strains shared a 98.5–99% nucleotide identity with the previously documented KX173339, KX173338, KX173340, and KY038016 Chinese strains [37].

In summary, since 2014, an increased number of outbreaks of vesicular disease caused by SVA have been reported worldwide, affecting swine in the United States [38,39] and other major swine-producing countries across the world, including China [31], Colombia [35], Thailand [36], Vietnam [37], and Brazil [40].

SVA causes a vesicular disease that is undifferentiated from the clinical signs of FMD, swine vesicular disease, vesicular stomatitis, or vesicular exanthema [18,39].

Macroscopically, lesions consist of different sizes, mostly of a few millimeters, intact and ruptured fluid-filled vesicles localized in the coronary band, carpus, the snout, and the tongue (Table 1) [18,41,42,43]. Consequently, affected adult pigs often present lameness, anorexia, lethargy, and fever (40.3–40.8 °C) [27].

**Figure 1:**
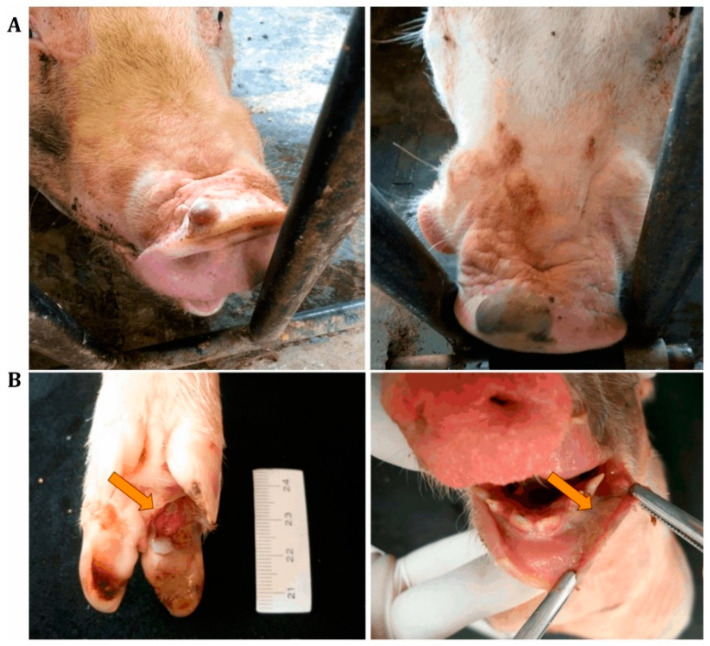
Lesions associated with senecavirus infection. (**A**) Fluid-filled vesicles on the snouts of senecavirus-positive sows. (**B**) Ulcerative lesions on the foot of a three-day-old piglet (**left**) and ulcerative and necrothizing gengivitis in a one-day-old piglet (**right**), both positive for senecavirus [18].

When vesicles undergo rupture deep ulceration, crusting and necrosis are commonly observed in the interdigital space as well as multifocal ulceration of the skin on the snout and ulcerative glossitis of the tongue (Figure 1) [18,41,42,43].

As in other vesicular diseases, the ulcers begin to repair in 7 days and regeneration of the epithelium is usually complete within 2 weeks [44].

Histologically, epidermal hyperplasia with orthokeratotic and parakeratotic hyperkeratosis, ulceration, and infiltration of neutrophils are common findings in affected skin [23]. In the first 3–7 days after experimentally infecting finisher pigs, mild atelectasis and congestion lungs were observed while the lymphoid organs, such as tonsils, lymph nodes, and spleen, showed hyperplasia. Of note, multifocal dermal separation with inflammatory cell infiltration, hemorrhage, and fibrin accumulation were noticed [39]. Other microscopic lesions have not been constantly observed in further tissues of affected animals [23].

In ETNL associated with SVA, piglets showed weakness, lethargy, excessive salivation, cutaneous hyperemia, neurological signs, diarrhea, and death [24]. These clinical signs persisted for 3–10 days before ending in surviving piglets. The most frequent gross lesions reported by Leme et al. 2016 in ENTL piglets [44] were ulcerative lesions in the tongue and coronary bands and kidney hemorrhages, while histologically, interstitial pneumonia was frequently recorded followed by diphteric glossitis, myocarditis, ballooning degeneration of the transitional epithelium of the urinary bladder and the ureters, and lymphoplasmacytic encephalitis. Interestingly, the transitional epithelium of the renal pelvis and of the urinary bladder, ependymal cells of the choroid plexus, the tongue epithelium, and the enterocytes of the small intestine were stained with SVA monoclonal antibodies to detect SVA by immunohistochemistry [44].

In 2017, Oliveira and coauthors [45] conducted a comprehensive study on 1 to 10-day-old piglets from 23 farms of South and Southeast regions in Brazil, with clinical manifestations indicative of ETNL, including diarrhea, neurological signs, reduced weight gain, and sudden death. As previously described by Leme et al., 2016 [44], the most frequent gross lesions were renal petechial hemorrhage, rib impressions on the lungs’ pleural surface, pulmonary edema, and congestion. In addition, there were cases of ulcerative lesions at the coronary band, mesocolonic edema, vesicles at the snout, and lymphadenopathy. Less frequently, lesions included Peyer’s patch hyperplasia, ulcerative glossitis, gingivitis and cheilitis, carpus skin abrasion, and hoof ulcerative lesion. Furthermore, vesicles in the muzzle with ulcerative lesions at the coronary band were observed in 21% of the piglets investigated.

Ballooning degeneration of the urinary bladder and of the renal pelvis transitional epithelium villous atrophy of the small intestine, interstitial pneumonia, and moderate, necrotizing dermatitis in the coronary bands, snout, metacarpal, and hoof were the main histopathological observed lesions [45]. Of note, rare intracytoplasmic, 6–7 µm in diameter, eosinophilic, structures suggestive of viral inclusion bodies of SVA were observed in the ballooning degenerated epithelium of the urinary bladder and within neurons in areas of nonsuppurative meningoencephalitis [45].

Recently, Liu and coauthors in 2021 [46], in a study on the pathogenicity analysis of 28-day-old weaned piglets challenged with an emerging SVA named CH/FuJ/2017, mentioned that piglets suffered from hemorrhage and inflammatory cell infiltration predominantly observed in the lung, liver, heart, and small intestine along hoof fluid-filled vesicles.

The diagnosis of SVA is based on clinical signs, detection of the virus, and antibodies against SVA [47].

As reported in recent work by Houston et al. (2020) [48], the diagnosis can be achieved by PCR and qRT-PCR virus isolation from vesicular material [42,49,50,51,52,53,54,55,56,57], in situ hybridization (ISH) [39,42,58], and immunohistochemistry (IHC) [44,45,46,47,48,49,50,51,52,53,54,55,56,57,58,59,60,61]. The detection of the nucleic acid of SVA by conventional and quantitative RT-PCR is regarded as the gold-standard test for etiological diagnosis and is considered a fast, sensitive, and specific method with numerous different viral targets reported [48]. Moreover, as pointed out by Leme and colleagues in 2017 [18] and by Gimenez-Lirola in 2016 [47], the variations in the viral shedding due to the progression of SVA infection suggested multiple diagnostic specimen analysis, with the tonsils and lymph nodes being positive after the viremic phase [48].

Antibody detection methods available consist of indirect immunofluorescence (IF), virus neutralization assays, competitive enzyme-linked immunosorbent assays (cELISAs), and indirect ELISAs targeting different structural proteins, including VP1, VP2, and VP3 [39,41,42,47,59,60]. As reported by Gimenez-Lirola and colleagues in 2016 [47], VP1 indirect ELISA provided a diagnostic sensitivity and specificity of 93% and 99%, VP2 ELISA yield 94.2% sensitivity and 89.7% specificity while VP3 protein showed minimal immunoreactivity, based on ROC analysis [59]. Moreover, the authors demonstrated that viral shedding is present also in animals without clinical disease with SVA-specific IgG response, suggesting that the virus may be circulating subclinically [47]. SVA diagnosis can also be accomplished by the ISH-RNAscope technique, targeting a complementary nucleotide sequence of the SVA (301–345 region of VP1 gene, GenBank: EU271758.1) [58], and IHC with monoclonal antibodies [44,61].

Recently, a reverse-transcription loop-mediated isothermal amplification (RT-LAMP) [62], a recombinase polymerase amplification (RPA) integrated with lateral flow (LF) distrips [63], and a fluorescent hydrolysis probe-based insulated isothermal PCR (iiPCR) has been developed for a quick and low-cost diagnosis of SVA [64].

## 3. *Torque teno sus virus* (TTSuV)

*Torque teno virus* (TTV) is a small, non-enveloped virus with a single-stranded negative-sense circular DNA genome, belonging to the family *Anelloviridae* [64,65]. The TTV’s genome size varies from 2.0 kb to 3.9 kb, depending on the host species [65]. The *Anellovirus* genus comprises viruses that infect various animal hosts, such as pigs (*Iotatorquevirus* and *Kappatorquevirus*), cats (*Etatorquevirus*), dogs (*Thetatorquevirus*), and tupaia (*Deltatorquevirus*) [66,67]. TTV infecting swine and wild boars has been named as *Torque teno sus virus* (TTSuV). Nowadays, the genus *Iotatorquevirus* with the species TTSuV1a and the *Kappatorquevirus* genus, which includes *Torque teno sus virus* *κ2a* species (TTSuVκ2a) and *Torque teno sus virus* *κ2b,* are approved by the International Committee on Taxonomy of Viruses [66,67].

The TTSuV genome is approximately 2.8 kb in length, comprising a highly conserved untranslated region (UTR) and at least five gene products, with ORF1 encoding the viral capsid protein, ORF2 encoding a non-structural protein essential for virus replication, and suppression of the NF-kB pathway and ORF3 of unknown function [64,68,69].

Generally, Anellovirus spreads horizontally, mainly via the fecal–oral route [70,71], while vertical and transplacental/intra-uterine are also important methods of transmission. Adding to this, *Torque teno sus* viruses may also be found in the semen of boars, suggesting the sexual route as an important transmission route, even if it has not been reported that the virus can affect the qualitative and quantitative features of the semen itself [72]. Due to the mentioned transmission routes, TTSuVs are classified as ubiquitous viruses with a worldwide distribution [71,73,74].

Furthermore, it has not yet been established whether TTSuV infection causes a specific disease as the primary agent, as TTSuV infections are common in both healthy and diseased swine [14].

At present, no clinical signs are specifically associated with TTSuV infection [75,76].

*Torque Teno Sus virus* was first reported in 1999 [77], even though it was shown to have been circulating in pig farms since 1985 [78].

TSuV1 and TTSuVk2a have been detected in pig populations worldwide with frequencies of infection ranging from 24 to 100% [79,80,81,82,83,84,85,86], while very little data are reported in wild boars worldwide [72,74,82,87,88].

On the other hand, TTSuVk2b has been recently detected in pig sera from many countries, with infection rates varying from 0 to 100% [83].

In Europe, the TTSuV1 DNA prevalence has been high in serum, being 66–76% in Spain [89,90], 77% in Sweden [91], 71.4% in Slovakia [92], and 62.3 in Italy [93].

Several studies have suggested that coinfection of *Iotatorquevirus* species may act as a cofactor or trigger the development of disease caused by other porcine pathogens [14,70,82,94,95]. TTSuVs have been detected in combination with economically important swine viral pathogens, such as porcine circovirus type 2 (PCV2), porcine parvovirus (PPV), and porcine reproductive and respiratory syndrome virus (PRRSV) and could influence the development of postweaning multisystemic wasting syndrome and porcine dermatitis and nephropathy syndrome [70,84,89,90,91,94,95].

TTSuV DNA has been found in various porcine tissues, including brain, bone marrow, heart, liver, lungs, lymph nodes, kidneys, and spleen, indicating a tropism for multiple tissues [14,70,86,90].

Studies conducted to specify the gross findings and histopathological manifestations of TTSuV are very limited and the attribution of lesions solely to TTSuV infection is still unclear (Table 2) [86,96,97,98].

In a study performed on gnotobiotic pigs with an experimental TTSuV1 infection, Krakowka and Ellis (2008) described a severe, progressive, and time-dependent, interstitial pneumonia, membranous glomerulonephropathy, and a moderate lymphohistiocytic infiltrate in the liver as well as transient thymic atrophy [97].

Mei and coauthors (2011)*,* in a study on the histopathological investigation in porcine experimental infection with TTSuV2, reported no obvious gross lesions, while several microscopic lesions, including hyperemia and congestion, in the myocardium and endocardium, interstitial pneumonia, membranous glomerular nephropathy, and mild inflammatory cell infiltration in portal areas of the liver were detected in the infected animals [98].

Recently, Polster and colleagues (2022) reported additional lesions in TTSUV1-positive animals, including a non-suppurative encephalitis, and in a few examined cases, a mild lymphoid depletion, follicular hyperplasia in spleen and mesenteric lymph node, non-suppurative myocarditis, chronic interstitial nephritis, hepatitis, and catarrhal to suppurative bronchopneumonia or bronchial-interstitial pneumonia [86].

Molecular testing of TTSuV can be performed on serum samples by conventional nested polymerase chain reaction (PCR) and real-time PCR [73,98,99,100] as well as in the liver, spleen, heart, tonsils, and in normal blood of stillborn fetuses [90]. Krakowka and Ellis, in 2008 and, recently, Polster and colleagues (2022), reported the TTSuV1 DNA probe for ISH [86,97].

## 4. Lateral Shaking Inducing Neuro-Degenerative Agent (LindaV)

Pestiviruses are small, single-stranded RNA genome viruses, belonging to the family *Flaviviridae,* genus *Pestivirus* [101,102]. In 2017, the last update of pestivirus taxonomy resulted in the classification of 11 species, designated *Pestivirus A* through *Pestivirus K* [94]. In particular, the classical species from *A* to *D* included Bovine viral diarrhea virus 1 and 2 (BVDV1, BVDV2), Classical swine fever virus (CSFV), and Border disease virus (BDV), respectively. Furthermore, the species *E* to *K* comprise pronghorn antelope pestivirus (PAPeV), porcine pestivirus (PPeV), giraffe pestivirus (GPeV), HoBi-like pestivirus (HoBiPeV), Aydin-like pestivirus (AydinPeV), rat pestivirus (RPePV), and atypical porcine pestivirus (APPeV) [102]. Moreover, recently, Postel and colleagues (2021) in the “proposed update to the taxonomy of Pestiviruses”, added eight additional species within the genus, including pathogens associated with disease in pigs or small ruminants [103].

Linda virus was proposed as *Pestivirus L* based on the discovery in 2015 of a novel pestivirus in a piglet farm in Styria, Austria. During this outbreak, piglets exhibited severe lateral shaking and were unable to suck milk, with elevated preweaning death rates. The newly discovered virus was provisionally termed lateral shaking inducing neuro-degenerative agent and caused severe hypomyelination in the white matter of the spinal cord inducing congenital tremors (CT) (Table 3) [104]. The genome sequence (GenBank NC035432) reveals that LindaV is genetically related to another newly discovered pestivirus, termed Bungowannah virus (BuPV, *Pestivirus F*), which was isolated from diseased pigs in a large pig holding in Australia in 2005 [105]. Of note and differently from LindaV, BuPV cannot induce congenital tremor [105].

So far, epidemiological studies performed in Germany and Switzerland regarding the prevalence of LindaV RNA in porcine serum samples tested negative [106,107].

Interesting, infections of immunocompetent piglets with LindaV result in temporary viremia with a quick seroconversion, although the virus persisted in the tonsil and lymphoid organs and was still detectable after 21 days [108]. However, no clinical signs nor macroscopic nor histological lesions were observed in challenged animals [108].

Up to now, LindaV has been detected with a 0.7% seroprevalence at the farm level in Austria [109]. Interestingly, clinical signs of congenital tremors had never been observed on that farm, even if a novel, genetically related LindaV strain (LindaV strain Austria2) was isolated [109].

Recently, Kiesler and colleagues reported a novel LindaV strain (LindaV strain Austria3) in a farrow-to-finish farm in Carinthia, Austria. The authors described reproductive disorders in sows and gilts (i.e., abortions, neonatal deaths, stillborn, and mummified piglets) followed by signs of congenital tremors in 20 litters [110]. No specific gross lesions were observed in examined organs, while multifocal scattered and perivascular lymphoplasmacellular encephalitis and myelitis (Table 3). Furthermore, LindaV was detected in the cytoplasm of neurons by IHCusing pestivirus E2-specific monoclonal antibody, but not related to inflammatory lesions [110].

Based on the reported literature and the detection method mostly based on LindaV-Specific RT-qPCR Assay, Linda V seems to be confined in Austria, while no other outbreaks have been reported in other locations [104,108,109,110].

## 5. Conclusions

Emerging swine viral diseases continue to challenge the global swine population. Despite the majority of these viruses being omnipresent in swine populations, the limited knowledge of the pathogenesis and related gross and histological lesions limited the likelihood of the development of data-based control strategies aimed to limit the potentially great impact on the swine industry. In this paper, we reviewed and summarized the main pathological findings related to *Senecavirus A*, *Torque teno sus virus*, and Linda virus, suggesting a call for further multidisciplinary studies aimed to fill this lack of knowledge and better clarify the potential role of those viral diseases.

## Figures and Tables

**Table 1:** Main scientific studies describing the gross and histological lesions and diagnostic methods applied for *Senecavirus A* identification.

Study	Gross Lesions	Histopathology	Diagnostic Method(s)
Plasma et al., 2008 [22]	Coalescing erosions or ruptured vesicle on the snout and coronary band.	NA *	PCR
Singh et al., 2012 [23]	Intact and ruptured vesicles and erosions in the oral cavity, around the nares and the coronary bands. Ulcers in fore and hind limbs.	Suppurative and ulcerative dermatitis	RT-PCR
Vannucci et al., 2015 [24]	Vesicles and coalescing erosions on the snouts and coronary bands of sows. Occasionally, vesicles and erosions on the coronary bands of piglets.	Hyperkeratosis, intra- and inter-cellular edema of keratinocytes and acantholytic degeneration of basal keratinocytes	Next-generation sequencing, RT-PCR
Baker et al., 2016 [27]	Nasal, coronary band, and hoof vesicular lesions in sows. No gross lesions in neonatal pigs.	NA	NA
Canning et al., 2016 [28]	Vesicular lesions on snouts and hoof lesions in sows. No gross lesions in neonatal pigs.	NA	RT-PCR
Wang et al., 2016 [29]	Ruptured vesicular lesions on the snout and coronary bands in sow	NA	RT-PCR
Wu et al., 2017 [31] *	Fluid-filled vesicles on the snout, interdigital cleft, coronary band. Petechial hemorrhages of kidney and interstitial pneumonia in piglets	Suppurative inflammation in dermis and epidermis, cell necrosis and damage of epithelial cells in hoof. Other lesions described are not strictly associated with SVA	RT-PCR
Montiel et al., 2016 [41] *	Intact or ruptured vesicular lesions on the coronary bands of toes and dewclaws or the interdigital space. Vesicular lesions and erosions on snouts.	NA	RT-PCR
Leme et al., 2016 [44]	Rib impressions on the pleural surface, pulmonary oedema, congestion of meningeal vessels, petechial hemorrhages of the kidney, diphtheritic glossitis, ulcerative lesions at the coronary band, multifocal cutaneous crusts	Interstitial pneumonia, lymphoid depletion, lymphocytic myocarditis, ballooning degeneration of transitional epithelium of the urinary bladder and ureters, lymphoplasmacytic encephalitis, vacuolation and atrophy of intestinal villi.	RT-PCR, IHC
Olivera et al., 2017 [45]	Erosive lesion at the coronary band, mesocolonic edema, multifocal ulceration of the skin	Ballooning degeneration of transitional epithelium, nonsuppurative meningoencephalitis, plexus choroiditis, atrophic enteritis	RT-PCR, IHC

* experimental infection; NA: not available; RT-PCR: real-time PCR; IHC: immunohistochemistry.

**Table 2:** Scientific studies describing the gross and histological lesions and diagnostic methods applied for *Torque teno sus virus* identification.

Study	Gross Lesions	Histopathology	Diagnostic Method(s)
Krakowka and Ellis, 2008 * [97]	Interstitial pneumonia, mild thymic atrophy, edema in the ventral region of the neck and thoracic mediastinum	Interstitial pneumonia, membranous glomerulonephropathy, moderate lymphohistiocytic infiltrate in the liver.	PCR, nPCR, ISH
Mei et al., 2011 [98] *	Limited and still unclear	Hyperemia and congestion in the myocardium and endocardium, interstitial pneumonia, membranous glomerular nephropathy, and lymphocytic inflammation in the liver.	nPCR, ELISA
Polster et al., 2022 [86]	NA	Non-suppurative encephalitis/meningoencephalitis/plexus choroiditis.	RT-PCR, ISH, IHC

* experimental infection; nPCR: nested PCR; ELISA: enzyme-linked immunosorbent assay; RT-PCR: real-time PCR; ISH: in situ hybridization; IHC: immunohistochemistry.

**Table 3:** Scientific studies describing the gross and histological lesions and diagnostic methods applied for Linda virus identification.

Study	Gross Lesions	Histopathology	Diagnostic Method(s)
Lamp et al., 2017 [104]	No specific gross lesions	Hypomyelination in the white matter of the spinal cord	RT-PCR, IHC
Kiesler et al., 2022 [110]	No specific gross lesions	Brain and spinal cord perivascular lymphoplasmacellular infiltrations	RT-PCR, IHC

RT-PCR: real-time PCR; IHC: immunohistochemistry.

## Data Availability

Not applicable.
